# Involvement of COUP-TFs in Cancer Progression

**DOI:** 10.3390/cancers3010700

**Published:** 2011-02-18

**Authors:** Antoine Boudot, François Le Dily, Farzad Pakdel

**Affiliations:** Molecular and Cellular Interactions, UMR CNRS 6026, IFR 140 GFSA, University of Rennes 1, Rennes, France; E-Mails: f_ledily@yahoo.fr (F.L.D.); antoine.boudot@univ-rennes1.fr (A.B.)

**Keywords:** orphan nuclear receptors, COUP-TFs, cancer progression, steroid hormones, cross-talk

## Abstract

The orphan receptors COUP-TFI and COUP-TFII are members of the nuclear receptor superfamily that play distinct and critical roles in vertebrate organogenesis, as demonstrated by loss-of-function COUP-TFI and/or COUP-TFII mutant mice. Although COUP-TFs are expressed in a wide range of tissues in adults, little is known about their functions at later stages of development or in organism homeostasis. COUP-TFs are expressed in cancer cell lines of various origins and increasing studies suggest they play roles in cell fate determination and, potentially, in cancer progression. Nevertheless, the exact roles of COUP-TFs in these processes remain unclear and even controversial. In this review, we report both *in vitro* and *in vivo* data describing known and suspected actions of COUP-TFs that suggest that these factors are involved in modification of the phenotype of cancer cells, notably of epithelial origin.

## 1. Introduction

**Figure 1.**
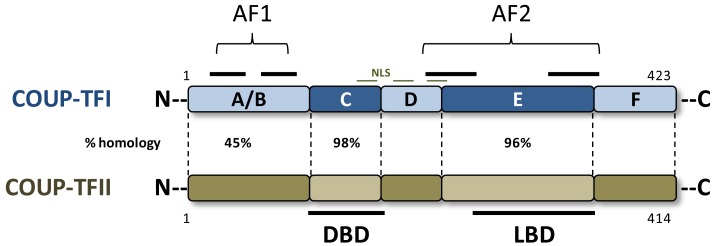
Schematic structure of human COUP-TFI and COUP-TFII proteins. The different domains are represented with the percentage of amino acid identity between the two receptors. The N-terminal and C-terminal transactivation functional domains (AF-1, AF-2); as well as DNA-binding domain (DBD); nuclear localization signal (NLS); and putative ligand-binding domain (LBD) are shown.

Chicken-Ovalbumin-Upstream-Promoter-Transcription Factors (COUP-TFs) were first characterized as fundamental transcription factors for chicken ovalbumin gene expression [1,2]. Two major homologues have been described in vertebrates, named COUP-TFI and COUP-TFII [1,2]. These two factors belong to the nuclear receptors (NRs) super family. Therefore, they share the highly conserved modular structure characteristic of these ligand activated transcription factors, comprising a central DNA binding domain (DBD), a putative C terminal ligand binding domain (LBD), and two activation functions, namely AF-1 and AF-2, necessary for co-factor recruitment (Figure 1) [1]. However, no natural ligand has been characterized so far for COUP-TFI or COUP-TFII, although supra-physiological doses of retinoic acid (RA) induce structural activation of COUP-TFII LBD *in vitro* [3]. Thus, COUP-TFs are to date classified as orphan nuclear receptors. The orphan receptor Ear2 has been reported as a third member of the COUP-TFs family since this protein shares DNA binding activities and has the capacity to heterodimerize with COUP-TFI. However, Ear2 exhibits a higher divergence in protein sequence from the two other forms and we therefore focused on COUP-TFI and COUP-TFII [1,2,3].

These two NRs are encoded by two distinct genes located on human chromosome 5 and 15, respectively. Nevertheless, they display high homology in amino acid sequence suggesting conservation of a primordial duplication during evolution. Homology in sequence varies from nearly 100% in the DBD to about 45% for the N-terminal region (Figure 1). This suggests possible divergences in functions and/or regulation between the two factors. In addition, both orphan receptors are highly conserved during evolution since the human COUP-TFs share almost 95% homology with other vertebrate and invertebrate proteins. This suggests that these orphan NRs could be primordial members of the NR family [1].

In vertebrates, both forms of COUP-TFs are expressed during early development and have partially overlapping patterns of expression. However, COUP-TFI is mainly expressed in the developing peripheral nervous system (PNS) and central nervous system (CNS), while COUP-TFII is observed in the mesenchymal area of internal organs and shaping vasculature, suggesting their involvement in distinct developmental processes [2]. Homozygous deletions of COUP-TFI in mice generate many defects, particularly in the CNS, leading to perinatal lethality. A significant reduction of axonal projection and arborization as well as an excessive cell death in cortical layers were reported in COUP-TFI null mice, confirming the importance of this factor in neuronal development and differentiation. In contrast, null mutant mice for COUP-TFII die around E10 because of growth-retardation in the vasculature of the head, spine and heart, highlighting the crucial role of COUP-TFII in the regulation of mesenchymal-endothelial interactions and blood vessel formation during development [2].

Interestingly, there is much evidence suggesting that these orphan NRs are directly involved in various biological aspects including cell proliferation, survival, angiogenesis and cell migration at later stages in a wide range of tissues in adults. In addition, COUP-TFs have been described to modulate the activity of several other transcription factors known to be involved in carcinogenesis and cancer progression. This suggests that COUP-TFs may take part in many dysregulated biological functions and may be involved in modifications of cellular phenotype as it is observed during cancer progression.

## 2. Mechanism of Actions and Physiological Roles of COUP-TFs

Since no specific ligand has been characterized so far for these receptors, the mechanisms that allow the regulation of the activity of COUP-TFs are not totally understood. Nevertheless, two main mechanisms of control of their activity have been proposed. First, the level of expression of COUP-TFs can be controlled by several factors or signaling cascades, including retinoids [1,4], estradiol (E2) [5], sonic hedgehog [1], or mitogenic activated protein kinase (MAPK) pathways [6]. Secondly, COUP-TFs activity can be controlled by post-translational modifications. Several kinase pathways have been shown to target specific phospho-sites on the COUP-TFI protein. These phosphorylation events in turn modulate its transcriptional activity [7].

As members of the nuclear receptors family, COUP-TFs mainly act as direct regulators of transcription by binding to specific regulatory elements on the promoter of their target genes. Although, the consensus COUP-TF-binding site is a direct repeat of an AGGTCA motif separated by one nucleotide, COUP-TFs display great flexibility for DNA binding. Indeed, they bind a large variety of response elements which can be recognized by several other members of the NR family, including thyroid hormone receptor (TR), retinoic acid (RA) receptor (RAR), vitamin D receptor (VDR) or estrogen receptor (ER) [1,8–10]. As a consequence, COUP-TFs interfere with NR signaling, acting mainly as a repressor of their activity on transcription via competition for occupancy of common DNA binding sites. Further, COUP-TFs are able to modulate NR signaling by other mechanisms: sequestration of the retinoid X receptor (RXR) by direct interaction with this universal partner, direct interaction with transcriptional cofactors, and controlling histone acetylation status and chromatin compaction [10]. Thus, hormonal regulation of many target genes as well as cellular response to external signals may be altered by excessive expression of these orphan receptors. This wide range of action of COUP-TFs may explain the importance of these factors in the regulation of key biological processes during development. Although the transcription of several genes has been described to be modulated *in vitro* by direct or indirect action of COUP-TFs, the list of endogenous and specific COUP-TF-target genes is far from exhaustive. However, an interesting study has recently identified numerous candidate COUP-TFI target genes, using genome-wide microarray analysis and a bioinformatics approach [11].

### 2.1. COUP-TFs Are Involved in the Orientation of Cellular Differentiation

COUP-TFs are mainly expressed in dedifferentiated cells such as mesenchymal cells, not in terminally differentiated epithelia [1]. Actually, COUP-TFII clearly prevents cell differentiation in many tissues. For example, COUP-TFII is generally down regulated during myogenic differentiation. When over-expressed in myoblasts, COUP-TFII blocks the induction of *myoD* and *myogenin*, two genes involved in the differentiation process of these cells [12,13]. In addition, COUP-TFII is also down regulated during adipocyte differentiation. Overexpression of this NR or physiological induction by Hedgehog and Wnt pathways leads to a blockage of cell differentiation due to a decreased expression of activators of the adipogenic program. These factors include Kruppel-like factor 15 (KLF15), sterol regulatory element binding transcription factor 1 (SREBP1c), Peroxisome proliferator-activated receptor (PPAR) γ1, PPARγ2, CCAAT/enhancer binding protein-α (C/EBPα) and early B-cell factor 2 (EBF2) [14,15]. Mechanistically, the repression of PPARγ gene expression by COUP-TFII was explained by the recruitment of nuclear corepressors (SMRT and NCoR) near to the PPARγ gene promoters. These corepressor complexes maintain a hypoacetylated and repressed state of the chromatin [15].

Furthermore, the link between COUP-TFs and differentiation has been highlighted by their role in the control of a specific neuronal-differentiation program mediated by retinoids. COUP-TFs are indeed regulators of the RA signaling pathway that contributes to specify positional identity. As an example, RA induces Cdx1, a member of the caudal-related homeobox transcription factor gene family, during embryonic development. In the anterior area of the embryo, this RA-induction of Cdx1 is antagonized by COUP-TFs that is itself induced by RA. Hence, this COUP-TFII effect on RA signaling contributes to the patterning of vertebral anterior-posterior axis [16]. Nevertheless, COUP-TFs seem to play a dual role in RA signaling during development. For instance, COUP-TFI reduces retinoid-induced growth arrest in mouse embryonic stem cells, but enhances RA-induced extra embryonic endoderm differentiation [17]. Furthermore, although COUP-TFI has been reported to contribute to Oct-4 (a stem cell-specific factor) repression during P19 cells differentiation induced by RA [18], overexpression of the two forms of COUP-TFs antagonized *in vitro* RA-induced neuronal differentiation in PCC7 [19] and P19 cell lines [20], as in *Xenopus* embryos [21]. Altogether, these results suggest that the level of COUP-TFs expression might be essential for their action in RA signaling.

### 2.2. COUP-TFs Are Involved in the Control of Cell Growth

COUP-TFI null mice exhibit abnormal morphology during development of the cortical area and the PNS, due to excessive apoptosis in these regions, suggesting that COUP-TFI is involved in cell survival [22,23]. In addition, during the development of postnatal mouse cerebellum, COUP-TFII expression is involved in brain size increase, by inducing proliferation and reducing apoptosis of granule cells precursors. The direct regulation of the growth factor IGF-1 (insulin like growth factor 1) by COUP-TFII in Purkinje cells is responsible for these proliferative and anti-apoptotic signals [24].

Seven-up (svp), the homolog of COUP-TFs in *Drosophila*, is a downstream target of epithelial growth factor receptor (EGFR) signaling [25]. The induction of svp by the EGFR signaling cascade consequently regulates the transcription of cell cycle genes to promote proliferation of malpighian tubules' cells. A similar mechanism of COUP-TFII regulation by EGFR signaling was also observed in human cell lines [6]. However, the authors did not check if this regulation of COUP-TFII could play a role in the proliferative effect of this growth factor's signaling in mammalian cells. Nevertheless, treatment with anti-proliferative agent such as OSM (Oncostatin M) resulted in a radical reduction of COUP-TFII expression.

Na^+^/H^+^ exchanger (NHE), a mammalian plasma membrane protein, is another downstream target of COUP-TFs in 3T3 fibroblast cell lines and P19 cells, which is known to promote cell proliferation [26]. Both subtypes of COUP-TF directly bind as homodimers and heterodimers to the proximal promoter of the *NHE1* gene, increasing its expression.

Although, the expression of COUP-TFs seem to be correlated with cell growth induction, *in vivo* studies also reported that COUP-TFs could be associated with repression of cell proliferation. COUP-TFI coordinates the expression of several factors whose signaling networks are fundamentals for the balance between proliferation and differentiation. For instance, fibroblast growth factor (FGF) signaling, which leads to activation of FGFR tyrosine kinase receptors, is repressed by ectopic expression of COUP-TFI and accentuated by depletion of this NR during cerebral cortex formation [27]. Repression of FGF signal attenuates MAPK/ERK, AKT, and β-Catenin signaling and consequently reduce cell proliferation [28]. In addition, Notch pathway is induced by COUP-TFI expression in hair cells, supporting cell cycle exit and cell differentiation [29].

Spatio-temporal changes of the expression of these orphan receptors together with changes of the activity of other signaling pathways in a cell-context dependent manner may explain these apparent dual actions on cell proliferation.

### 2.3. COUP-TFs Are Involved in Cell Migration Control

In addition to the control of growth/differentiation during CNS development, one of the major contributions of COUP-TFs and particularly COUP-TFI, is to promote neurogenesis and axon arborization [10,22,23]. The involvement of COUP-TFs in these processes is mainly linked to their action in the control of the migratory capacities of neurons during brain development. Kanatani and collaborators described that COUP-TFII expression is sufficient to induce migration behavior and specific orientation of interneurons migration toward the caudal region of the brain [30]. Furthermore, ectopic expression of COUP-TFI or COUP-TFII enhances cells migration in mouse embryos CNS [31]. Specific downstream targets of COUP-TFs and the mechanisms of regulation of migration are still mainly unknown. However, COUP-TFI has been shown to regulate expression of various factors implicated (i) in neuron morphogenesis and axon outgrowth such as cytoskeleton polymerizing factors [32] and (ii) in cell adhesion proteins, such as extra cellular matrix (ECM) components and notably vitronectin [20]. It is interesting to note that COUP-TFI also represses *in vitro* expression of type VII collagen in human cell lines [33]. Moreover, in fibroblast cells, cell contact stability has also been reported to be affected by COUP-TFI overexpression which induces changes in the expression of cell attachment proteins [34].

### 2.4. COUP-TFII Is Related to Angiogenesis

COUP-TFII null mice die as a result of hemorrhage and edema in the brain and heart because of malformations or lack of vasculature in some regions, indicating that this NR is involved in blood vessel formation [35]. Indeed, COUP-TFII is a key regulator of vascular remodeling as well as in primitive lymphatic vessel formation since it controls the vascular endothelial growth factor (VEGF) system expression and signaling: VEGF-C, VEGF-D and VEGF receptor (VEGFR-3). COUP-TFII expression in vascular endothelial cells supports endothelial differentiation, regulating neuropilin-2, a coreceptor for VEGF-C [36] and Prox1, a transcription factor necessary for VEGFR-3 expression [37]. Moreover, in mesenchymal cells, COUP-TFs directly regulate the expression of the pro-angiogenic factors VEGF-D [38] and are supposed to control the expression of Angiopoietin-1 gene [35]. Therefore, they participate in the mesenchymal-endothelial interactions during the process of angiogenesis.

**Figure 2.**
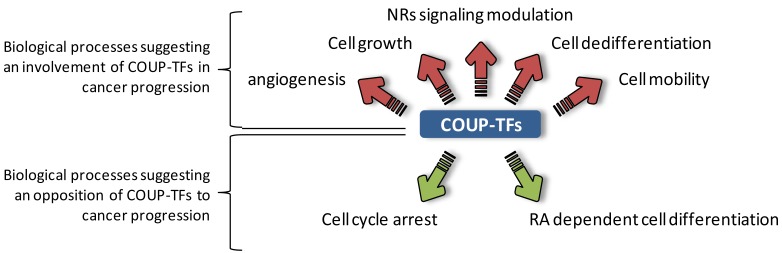
Principal biological processes controlled by COUP-TFs during embryogenesis that suggest a role of these factors during cancer progression. Biological processes that could promote cancer progression are linked by red arrows, while those that could promote regression of cancer are connected by green arrows. During embryonic development, COUP-TFs are key regulators of various processes that are also linked to cancer progression. Several downstream targets of these factors are connected to angiogenesis, cell growth or mobility which could be linked to cancer cell growth and metastatic potential. Moreover, both COUP-TFs are able to interfere with nuclear receptor signaling which are known to play critical roles during hormone-dependent cancers development. On the other hand, depending on the circumstance, COUP-TFs also control some biological aspects that could be associated with cancer regression. These orphan NRs were reported to control cell cycle arrest to support neuronal differentiation, or to contribute to RA dependent differentiation. NRs, nuclear receptors; RA, retinoic acid.

Most of the reported actions of COUP-TFs occur during embryonic development during which COUP-TFI and COUP-TFII have been shown to exert specific and redundant activities. Levels of expression of both COUP-TFs decrease after organogenesis but these receptors remains ubiquitously expressed in adults, suggesting that they still have a role at later stages of development in normal and potentially pathological biological processes. Indeed, re-activation of the embryonic network is often observed during cancer development, as illustrated by epithelial-mesenchymal-transition (EMT). The process of EMT, which consists of the loss of cell-cell contacts and polarized-epithelial characteristics for the acquisition of a fibroblastic and dedifferentiated-mesenchymal phenotype, is an essential step for carcinoma cells to metastasize [39,40]. The variety of COUP-TFs effects that we have described above suggest that these NRs could be involved in these events. For instance, COUP-TF's impacts on cell fate determination strongly suggest that these NRs may play a role in cancer progression by influencing the differentiation of cancer cells or by determining their proliferative behavior. COUP-TFs are proposed to control many biological functions involved in cell migration such as cell-cell contacts and adhesion as well as synthesis of the extracellular matrix (ECM) components. These processes are highly related to cancer progression, through modulation of metastatic potential of cancer cells. In addition, since angiogenesis is one of the fundamental steps of cancers progression, a direct connection between COUP-TFs and this biological process is in line with a contribution of these NRs in disease progression (Figure 2).

## 3. Potential Roles of COUP-TFs in Cancer Progression

**Figure 3.**
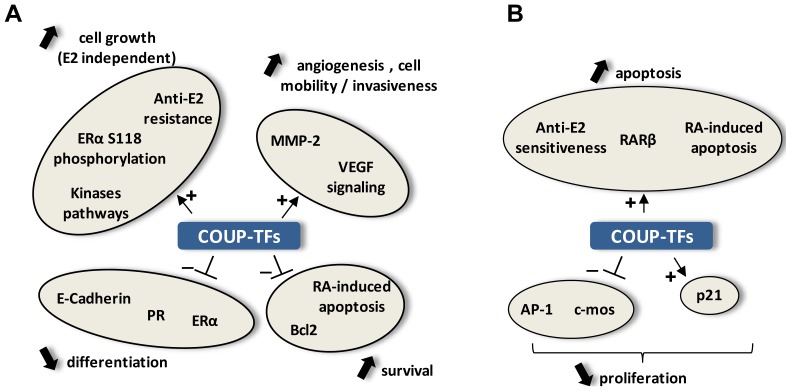
Identified-targets of COUP-TFs described in cancer progression or regression. COUP-TFs regulate positively (+) or negatively (−) the expression of various target genes or signaling pathways that could influence proliferation, survival or migration of cancer cells as well as differentiation state or angiogenesis of tumors. These contributions are linked to cancer progression (**A**) or cancer regression (**B**). MMP-2, matrix-metalo-protease-2; VEGF, vascular endothelial growth factor; RA, retinoic acid; ERα, estrogen receptor alpha; PR, progesterone receptor; Bcl2, B-cell CLL/lymphoma 2; RARβ, retinoic acid receptor beta; AP-1, activator protein-1; P21, cyclin-dependent kinase inhibitor 1A.

Several studies have analyzed the influence of orphan NRs on cancer cell phenotype and their potential involvement in cancer progression, modifications of cellular signaling pathways and cell response to treatments. Particularly, one of the principal actions of COUP-TFs may be the regulation of the activity of liganded NRs such as ER and RAR in the control of cell growth in hormone-dependent tumors. Depending on the cell type and the biological processes studied, COUP-TFs have been proposed to act positively or negatively on cancer progression (Figure 3). Notably, several studies suggest that, in line with their roles in cell fate determination, COUP-TFs can be involved in epithelio-mesenchymal transition, which is a critical event during oncogenesis. However, the exact role of COUP-TFs in generating the phenotype of cancer cells remains controversial.

### 3.1. Expression of COUP-TFs in Cancer Cell Lines

Several laboratories have reported diverse observations concerning the level of expression of COUP-TFs in established model cancer cell lines. Examination of the expression of COUP-TFs in cancer cell lines from different origins, from ovary [41], endometrium [42], breast [6,43,44] or lung [45], showed significant variations in COUP-TFs mRNA and protein levels. Kieback *et al.* demonstrated that COUP-TFI expression is associated with the dedifferentiation status of ovarian [41] and endometrial [42] cancer cells. COUP-TFI expression has also been proposed to be linked with the differentiated state of breast cancer cell lines, since its expression was correlated to dedifferentiated phenotypes of epithelial cells, notably low expression of the epithelial marker E-cadherin and expression of the mesenchymal marker vimentin [44]. Furthermore, results reported by More and collaborators suggested that COUP-TFII expression could be associated with loss of ER expression in mammary cancer cell lines [6]. The ER status represents an important indicator of hormone-dependent cancer differentiation state, ER-negative tumors being generally histologically less differentiated and exhibiting superior metastatic potential [46]. Ambiguously, a study from Nakshatri *et al.* showed that ER-positive breast cancer cells such as MCF-7, T47-D or ZR-75 cells express an elevated COUP-TFII expression, while ER-negative cells such as MDA-MB-231 and SkBr3 express a low level of this receptor [43].

Deviation between cancer cell lines may explain these discrepancies. However, the precise role of these factors in cancer progression remains unclear. In connection with the role of COUP-TFs in the control of migratory capacity during development, several groups analyzed the potential influence of these factors on cancer cell invasiveness. In lung carcinoma cancer cell lines, invasiveness correlates with COUP-TFII expression. Notably, COUP-TFII promotes anchorage independent cell growth and mobility by inducing the expression and activity of matrix metalloproteinases and focal adhesion kinase (FAK) [47]. Similarly, we observed that overexpression of COUP-TFI favors migration and invasion capacities of MCF-7 breast cancer cells [44]. Interestingly, these observations were correlated with the fact that the overexpression of COUP-TFI represses E-cadherin expression while it enhances the MAPK signaling pathway. Intracellular signaling pathways, FAK and MAPK cascades in particular, are known to be involved in the acquisition of mesenchymal characteristics, leading to EMT [48]. More *et al.* showed evidence that COUP-TFII expression correlates with EGFR signaling: COUP-TFII is induced by EGF treatment and is repressed by EGFR inhibitors [6]. These results have also suggested that when the MAPK pathway is constitutively activated, COUP-TFII reaches a maximum level of expression. Finally, in line with the role of COUP-TFs in angiogenesis during development, both COUP-TFI and COUP-TFII together with orphan receptor hepatocyte nuclear factor 4α (HNF-4α) are potent activators of VEGF-D expression in MKN-45 gastric carcinoma cell line [38] and in MCF-7 cell line [49], suggesting these factors impact cancer progression by promoting neo-angiogenesis and lymphangiogenesis.

Altogether, these results strongly suggest that increased expression of COUP-TFs in cancer cells is associated with the induction of the dedifferentiation phenotype, the acquisition of migration behavior, and the promotion of angiogenesis. All these events that could promote EMT, which is a crucial step for carcinoma cells to metastasize, may favor tumors to acquire more aggressive phenotypes. Interestingly, in a wide range of cancers, these processes are also regulated by other nuclear receptors, notably ER and RAR, of which activities are known to be modulated by the COUP-TFs.

### 3.2. Interference of COUP-TFs Expression with Estrogen Signaling

Estrogens are well recognized as steroid hormones that play pivotal roles in the mammary gland, where they act as key regulators of growth and differentiation of normal epithelium. In breast cancers, the ER status constitutes an important prognostic marker particularly for therapeutic value based on anti-estrogenic treatments. Moreover, ER-positive tumors are generally histologically more differentiated and show lower metastatic potential than ER-negative cancers [46]. Thus, interferences with estrogen signaling (modulation of E2-target genes expression; loss of ERα activity or expression) during breast tumorigenesis may result in the acquisition of invasive and metastatic properties, and therefore, in poor clinical outcomes.

Interference of COUP-TFs with ER signaling system, resulting from the modulation of gene expression, could lead to these phenotypic changes in breast cancer cells. However, the precise role of these factors in this process remains controversial.

ERα signaling can be repressed by COUP-TFI due to competition for DNA binding to an estrogen response element (ERE) [50–52]. On the other hand, COUP-TFI binding to DNA can also improve estrogenic signaling by synergistic or additional transactivations [53,54]. In addition, physical interaction between the two NRs can modulate ERα activity. On one hand, COUP-TFI/ER interaction disturb ERα binding to DNA leading to repression of E2 signaling [9]. On the other hand, interaction of ER with COUP-TFI can improve ERα transcriptional activity by increasing its affinity for MAPK, resulting in phosphorylation of ERα serine 118 [55]. Positive and negative influences of COUP-TFs could modulate E2 signaling or change the response to anti-E2 treatments in a way that depends on both promoter and cell contexts. In the context of hormonal-therapy, these precise regulations may induce the loss of hormone-dependent control of cancer progression.

For example, we recently reported that overexpression of COUP-TFI in E2-dependent breast cancer cells leads to a selective modulation of E2-sensitive gene expression, repressing or stimulating E2 induced response in a gene dependent way. Notably, COUP-TFI overexpression induces a reduction of progesterone receptor expression whereas it increases the expression of the well known anti-apoptotic E2-target gene *Bcl2* as well as the expression of the *Cathepsin* D (CTSD) gene. These changes in the pattern of expression of genes involved in the control of growth and apoptosis leads to a change in behavior of the cells from an E2-dependent to an E2-independent growth. Moreover, cell response to anti-estrogen is also altered since COUP-TFI overexpression induces resistance to 4-hydroxytamoxifen treatment [44].

However, the roles of COUP-TFs in the cellular response to anti-estrogenic compounds remain unclear. Although we observed that COUP-TFI may be involved in tamoxifen resistance of breast cancers; in contrast, Riggs *et al.* have proposed that high expression of COUP-TFII could be associated with increased tamoxifen sensitivity in breast cancer cells. Indeed, in this study, inhibition of COUP-TFII expression prevented the antiproliferative effects of tamoxifen treatment and increased MCF-7 cell basal proliferation [5].

### 3.3. Interference of COUP-TFs Expression with RA Signaling

Deregulation of RA signaling is associated with several pathologies, in particular cancer. Indeed, all trans-RA play major roles in the balance of cell proliferation and differentiation and synthetic RA-derivatives are commonly used in the prevention and treatment of different types of cancers. For instance, in primary lung tumors as well as in lung cancer cell lines, the expression of retinoic acid receptor β (RARβ) is significantly decreased. Moreover, blocking the expression of RARβ in transgenic mice by using antisense RARβ RNA favors the development of lung tumors. On the other hand, overexpression of RARβ into lung cancer cells suppresses their tumorogenicity in nude mice. Although, the molecular mechanisms underlying these effects are poorly understood, one explanation may be that RARβ represses the expression of genes encoding proliferative or angiogenic growth factors [56].

COUP-TFs modulate RA signaling, by acting mostly as negative regulators [57]. Different mechanisms of action have been described, such as competition for binding sites occupancy, competition for the universal heterodimeric partner RXR or active repression [10]. Since RA signaling in cancer cells is well documented to be correlated with cell cycle arrest and apoptosis induction, COUP-TFs action could thus stimulate cancer cells survival.

Accordingly, positive actions of COUP-TFs on tumor growth were reported in many types of cancers in response to RA. For instance, in salivary gland adenocarcinoma, COUP-TFI overexpression abolished RAR and RXR response to RA, and, as a result, protected cancer cells from apoptosis induced by RA treatment [58]. Sensitivity to RA, which promotes cell cycle arrest and apoptosis, can also be repressed by COUP-TFs expression in neuroendocrine-ACTH-secreting tumors [59].

Surprisingly, several groups observed reciprocal activities of COUP-TFs on RA modulation of cell growth. Notably, overexpression of COUP-TFs in lung cancer cells has been proposed to restore RA sensitivity [45]. COUP-TFI has also been shown to be required for induction of RARβ expression [60]. This study described a positive correlation between COUP-TFI expression and RARβ expression in various cancer cell lines including breast cancer, bladder cancer, and lung cancer cells. In MDA-MB231 breast cancer cells, the overexpression of COUP-TFI induced loss of anchorage-independent cell growth and enhanced the promoter activity of the RARβ gene. Inhibition of proliferation of MDA-MB-231 cells by COUP-TFII (but not COUP-TFI) overexpression has also been proposed by the inhibition of cdk2 activity through an elevation of p21 expression [43]. Furthermore, in cooperation with RARs, COUP-TFI has been reported to inhibit proliferation by inhibiting the activity of the AP-1 transcription factor [61].

### 3.4. *In Vivo* Observations

The few studies that analyzed the expression of COUP-TFs in tumors *in vivo* reported conflicting observations depending on the cancer type. *In vivo* immunochemistry assay targeting COUP-TFI revealed that breast epithelial cancer cells display much higher expression of COUP-TFI protein than that detected in normal breast epithelium [44]. The same observation was made regarding COUP-TFII expression [49]. COUP-TFII overexpression was furthermore associated with poor clinical outcome and invasive behavior of metastatic cells in lymph nodes. Because COUP-TFs, and particularly COUP-TFII, is involved in blood vessel formation, which is an essential step for tumor growth and progression, the authors proposed that the control of COUP-TFII in angiogenesis and lymphangiogenesis could be responsible for the poor clinical outcome [44]. In contrast, COUP-TFII expression has been reported to be down regulated in invasive epithelial ovarian tumors compared to human normal ovary tissue [62]. Hence, in ovarian cancer, COUP-TFI expression seems to be lower in malignant tissue than in benign or normal tissue [63]. Similar observations were reported in bladder transitional cell carcinoma [64]. These studies support the idea that loss of COUP-TFs expression could be linked to carcinogenesis. Finally, Shin *et al.* have proposed that increased expression of COUP-TFII in colorectal carcinomas is correlated to good clinicopathologic features and recommend the use of COUP-TFII as a biomarker for good prognosis in colorectal cancer [65].

These studies reported above surprisingly suggest that COUP-TFs could act as tumor promoter or suppressor depending on the cancer type and/or likely the differentiation state of the cancer cell. It is therefore conceivable that COUP-TFs have opposite and dual functions depending on the stage of cancer progression as it have been proposed for other factors. For instance, a recent study on P53 and TGFβ signaling showed that mutation of P53 changes the TGFβ activity from a tumor-suppressing form to a tumor-promoting form [66]. The roles of COUP-TFs in tumorigenesis would therefore depend on the expression of other factors or activity of signaling pathways in cancer cells and/or in the tumor microenvironment. Thus, further histological analysis of tumor samples including the involvement of the tumor microenvironment are needed to clarify these discrepancies.

## 4. Conclusions

Contribution of COUP-TFs to various developmental aspects, including cell differentiation, cell cycle and migration regulation, as well as their involvement in angiogenesis, suggests that these factors can also be involved in cancer progression. Moreover, impaired expression and/or activity of these orphan NRs could lead to abnormal transcriptional response of NRs, which could be at the source of cellular transformation due to irregular gene expression (Figure 2). In line with these possibilities, many studies revealed a direct impact of both COUP-TF subtypes on cancer progression through modulation of the capacity of cancer cells to proliferate, migrate or respond to external signals, including hormones (Figure 3A). Moreover, *in vivo* studies indicated that COUP-TFs are potent mediators of breast cancer progression, since high expression of COUP-TFs are associated with disease severity [44,49].

However, depending on the cellular context, possibly according to COUP-TF expression levels, or to other transcription factors and signaling pathways expression, COUP-TFs could act as promoters or inhibitors of phenotypical modifications during cancer progression (Figure 3B). Indeed, many different studies revealed that these transcription factors can also be related to a favorable prognosis of cancer progression and even propose the use of COUP-TFII as a biomarker for good prognosis [62–65].

Regarding the variety of endogenous targets of these orphan NRs and the various biological processes that they are involved in, further studies are needed to determine the exact contribution of COUP-TFs during tumorigenesis and cancer progression. Characterization of the molecular and cellular processes that contribute to patho-physiological activities of COUP-TFs may provide potential mechanistic explanations for drug resistance that will be useful in chemotherapy of cancer treatments.

## References

[1] Tsai S.Y., Tsai M.J. (1997). Chick ovalbumin upstream promoter-transcription factors (COUP-TFs): Coming of age. Endocr. Rev..

[2] Pereira F.A., Tsai M.J., Tsai S.Y. (2000). COUP-TF orphan nuclear receptors in development and differentiation. Cell. Mol. Life Sci..

[3] Kruse S.W., Suino-Powell K., Zhou X.E., Kretschman J.E., Reynolds R., Vonrhein C., Xu Y., Wang L., Tsai S.Y., Tsai M.J., Xu H.E. (2008). Identification of COUP-TFII orphan nuclear receptor as a retinoic acid-activated receptor. PLoS Biol..

[4] Clotman F., Van Maele-Fabry G., Picard J.J. (1998). All-*trans*-retinoic acid upregulates the expression of COUP-TFI in early-somite mouse embryos cultured *in vitro*. Neurotoxicol. Teratol..

[5] Riggs K.A., Wickramasinghe N.S., Cochrum R.K., Watts M.B., Klinge C.M. (2006). Decreased chicken ovalbumin upstream promoter transcription factor II expression in tamoxifen-resistant breast cancer cells. Cancer Res..

[6] More E., Fellner T., Doppelmayr H., Hauser-Kronberger C., Dandachi N., Obrist P., Sandhofer F., Paulweber B. (2003). Activation of the MAP kinase pathway induces chicken ovalbumin upstream promoter-transcription factor II (COUP-TFII) expression in human breast cancer cell lines. J. Endocrinol..

[7] Gay F., Barath P., Desbois-Le Peron C., Metivier R., Le Guevel R., Birse D., Salbert G. (2002). Multiple phosphorylation events control chicken ovalbumin upstream promoter transcription factor I orphan nuclear receptor activity. Mol. Endocrinol..

[8] Leng X., Cooney A.J., Tsai S.Y., Tsai M.J. (1996). Molecular mechanisms of COUP-TF-mediated transcriptional repression: evidence for transrepression and active repression. Mol. Cell. Biol..

[9] Klinge C.M., Silver B.F., Driscoll M.D., Sathya G., Bambara R.A., Hilf R. (1997). Chicken ovalbumin upstream promoter-transcription factor interacts with estrogen receptor, binds to estrogen response elements and half-sites, and inhibits estrogen-induced gene expression. J. Biol. Chem..

[10] Park J.I., Tsai S.Y., Tsai M.J. (2003). Molecular mechanism of chicken ovalbumin upstream promoter-transcription factor (COUP-TF) actions. Keio J. Med..

[11] Montemayor C., Montemayor O.A., Ridgeway A., Lin F., Wheeler D.A., Pletcher S.D., Pereira F.A. (2010). Genome-wide analysis of binding sites and direct target genes of the orphan nuclear receptor NR2F1/COUP-TFI. PLoS One.

[12] Muscat G.E., Rea S., Downes M. (1995). Identification of a regulatory function for an orphan receptor in muscle: COUP-TF II affects the expression of the myoD gene family during myogenesis. Nucleic Acids Res..

[13] Bailey P., Sartorelli V., Hamamori Y., Muscat G.E. (1998). The orphan nuclear receptor, COUP-TF II, inhibits myogenesis by post-transcriptional regulation of MyoD function: COUP-TF II directly interacts with p300 and myoD. Nucleic Acids Res..

[14] Xu Z., Yu S., Hsu C.H., Eguchi J., Rosen E.D. (2008). The orphan nuclear receptor chicken ovalbumin upstream promoter-transcription factor II is a critical regulator of adipogenesis. Proc. Natl. Acad. Sci. USA.

[15] Okamura M., Kudo H., Wakabayashi K., Tanaka T., Nonaka A., Uchida A., Tsutsumi S., Sakakibara I., Naito M., Osborne T.F., Hamakubo T., Ito S., Aburatani H., Yanagisawa M., Kodama T., Sakai J. (2009). COUP-TFII acts downstream of Wnt/beta-catenin signal to silence PPARgamma gene expression and repress adipogenesis. Proc. Natl. Acad. Sci. USA.

[16] Beland M., Lohnes D. (2005). Chicken ovalbumin upstream promoter-transcription factor members repress retinoic acid-induced Cdx1 expression. J. Biol. Chem..

[17] Zhuang Y., Gudas L.J. (2008). Overexpression of COUP-TF1 in murine embryonic stem cells reduces retinoic acid-associated growth arrest and increases extraembryonic endoderm gene expression. Differentiation.

[18] Schoorlemmer J., van Puijenbroek A., van Den Eijnden M., Jonk L., Pals C., Kruijer W. (1994). Characterization of a negative retinoic acid response element in the murine Oct4 promoter. Mol. Cell. Biol..

[19] Neuman K., Soosaar A., Nornes H.O., Neuman T. (1995). Orphan receptor COUP-TF I antagonizes retinoic acid-induced neuronal differentiation. J. Neurosci. Res..

[20] Adam F., Sourisseau T., Metivier R., Le Page Y., Desbois C., Michel D., Salbert G. (2000). COUP-TFI (chicken ovalbumin upstream promoter-transcription factor I) regulates cell migration and axogenesis in differentiating P19 embryonal carcinoma cells. Mol. Endocrinol..

[21] Schuh T.J., Kimelman D. (1995). COUP-TFI is a potential regulator of retinoic acid-modulated development in Xenopus embryos. Mech. Dev..

[22] Zhou C., Qiu Y., Pereira F.A., Crair M.C., Tsai S.Y., Tsai M.J. (1999). The nuclear orphan receptor COUP-TFI is required for differentiation of subplate neurons and guidance of thalamocortical axons. Neuron.

[23] Qiu Y., Pereira F.A., DeMayo F.J., Lydon J.P., Tsai S.Y., Tsai M.J. (1997). Null mutation of mCOUP-TFI results in defects in morphogenesis of the glossopharyngeal ganglion, axonal projection, and arborization. Genes Dev..

[24] Kim B.J., Takamoto N., Yan J., Tsai S.Y., Tsai M.J. (2009). Chicken Ovalbumin Upstream Promoter-Transcription Factor II (COUP-TFII) regulates growth and patterning of the postnatal mouse cerebellum. Dev. Biol..

[25] Kerber B., Fellert S., Hoch M. (1998). Seven-up, the Drosophila homolog of the COUP-TF orphan receptors, controls cell proliferation in the insect kidney. Genes Dev..

[26] Fernandez-Rachubinski F., Fliegel L. (2001). COUP-TFI and COUP-TFII regulate expression of the NHE through a nuclear hormone responsive element with enhancer activity. Eur. J. Biochem..

[27] Faedo A., Borello U., Rubenstein J.L. (2010). Repression of Fgf signaling by sprouty1-2 regulates cortical patterning in two distinct regions and times. J. Neurosci..

[28] Faedo A., Tomassy G.S., Ruan Y., Teichmann H., Krauss S., Pleasure S.J., Tsai S.Y., Tsai M.J., Studer M., Rubenstein J.L. (2008). COUP-TFI coordinates cortical patterning, neurogenesis, and laminar fate and modulates MAPK/ERK, AKT, and beta-catenin signaling. Cereb. Cortex.

[29] Tang L.S., Alger H.M., Pereira F.A. (2006). COUP-TFI controls Notch regulation of hair cell and support cell differentiation. Development.

[30] Kanatani S., Yozu M., Tabata H., Nakajima K. (2008). COUP-TFII is preferentially expressed in the caudal ganglionic eminence and is involved in the caudal migratory stream. J. Neurosci..

[31] Tripodi M., Filosa A., Armentano M., Studer M. (2004). The COUP-TF nuclear receptors regulate cell migration in the mammalian basal forebrain. Development.

[32] Armentano M., Filosa A., Andolfi G., Studer M. (2006). COUP-TFI is required for the formation of commissural projections in the forebrain by regulating axonal growth. Development.

[33] Calonge M.J., Seoane J., Massague J. (2004). Opposite Smad and chicken ovalbumin upstream promoter transcription factor inputs in the regulation of the collagen VII gene promoter by transforming growth factor-beta. J. Biol. Chem..

[34] Connor H., Nornes H., Neuman T. (1995). Expression screening reveals an orphan receptor chick ovalbumin upstream promoter transcription factor I as a regulator of neurite/substrate-cell contacts and cell aggregation. J. Biol. Chem..

[35] Pereira F.A., Qiu Y., Zhou G., Tsai M.J., Tsai S.Y. (1999). The orphan nuclear receptor COUP-TFII is required for angiogenesis and heart development. Genes Dev..

[36] Lin F.J., Chen X., Qin J., Hong Y.K., Tsai M.J., Tsai S.Y. (2010). Direct transcriptional regulation of neuropilin-2 by COUP-TFII modulates multiple steps in murine lymphatic vessel development. J. Clin. Invest..

[37] Yamazaki T., Yoshimatsu Y., Morishita Y., Miyazono K., Watabe T. (2009). COUP-TFII regulates the functions of Prox1 in lymphatic endothelial cells through direct interaction. Genes Cells..

[38] Schafer G., Wissmann C., Hertel J., Lunyak V., Hocker M. (2008). Regulation of vascular endothelial growth factor D by orphan receptors hepatocyte nuclear factor-4 alpha and chicken ovalbumin upstream promoter transcription factors 1 and 2. Cancer Res..

[39] Huang S., Ingber D.E. (2006). A non-genetic basis for cancer progression and metastasis: self-organizing attractors in cell regulatory networks. Breast Dis..

[40] Micalizzi D.S., Farabaugh S.M., Ford H.L. (2010). Epithelial-mesenchymal transition in cancer: parallels between normal development and tumor progression. J. Mammary Gland Biol. Neoplasi..

[41] Kieback D.G., Runnebaum I.B., Moebus V.J., Kreienberg R., McCamant S.K., Edwards C.L., Jones L.A., Tsai M.J., O'Malley B.W. (1993). Chicken ovalbumin upstream promoter transcription factor (COUP-TF): an orphan steroid receptor with a specific pattern of differential expression in human ovarian cancer cell lines. Gynecol. Oncol..

[42] Kieback D.G., Levi T., Kohlberger P., Fiedrich U., Press M.F., Rosenthal H.E., Mobus V.J., Runnebaum I.B., Tong X.W., Tsai M.J. (1996). Chicken ovalbumin upstream promoter—transcription factor (COUP-TF) expression in human endometrial cancer cell lines. Anticancer Res..

[43] Nakshatri H., Mendonca M.S., Bhat-Nakshatri P., Patel N.M., Goulet R.J., Cornetta K. (2000). The orphan receptor COUP-TFII regulates G2/M progression of breast cancer cells by modulating the expression/activity of p21(WAF1/CIP1), cyclin D1, and cdk2. Biochem. Biophys. Res. Commun..

[44] Le Dily F., Metivier R., Gueguen M.M., Le Peron C., Flouriot G., Tas P., Pakdel F. (2008). COUP-TFI modulates estrogen signaling and influences proliferation, survival and migration of breast cancer cells. Breast Cancer Res. Treat..

[45] Wu Q., Li Y., Liu R., Agadir A., Lee M.O., Liu Y., Zhang X. (1997). Modulation of retinoic acid sensitivity in lung cancer cells through dynamic balance of orphan receptors nur77 and COUP-TF and their heterodimerization. EMBO J..

[46] Sommer S., Fuqua S.A. (2001). Estrogen receptor and breast cancer. Semin. Cancer Biol..

[47] Navab R., Gonzalez-Santos J.M., Johnston M.R., Liu J., Brodt P., Tsao M.S., Hu J. (2004). Expression of chicken ovalbumin upstream promoter-transcription factor II enhances invasiveness of human lung carcinoma cells. Cancer Res..

[48] Moustakas A., Heldin C.H. (2007). Signaling networks guiding epithelial-mesenchymal transitions during embryogenesis and cancer progression. Cancer Sci..

[49] Nagasaki S., Suzuki T., Miki Y., Akahira J., Shibata H., Ishida T., Ohuchi N., Sasano H. (2009). Chicken ovalbumin upstream promoter transcription factor II in human breast carcinoma: possible regulator of lymphangiogenesis via vascular endothelial growth factor-C expression. Cancer Sci..

[50] Jiang J.G., Bell A., Liu Y., Zarnegar R. (1997). Transcriptional regulation of the hepatocyte growth factor gene by the nuclear receptors chicken ovalbumin upstream promoter transcription factor and estrogen receptor. J. Biol. Chem..

[51] Liu Y., Yang N., Teng C.T. (1993). COUP-TF acts as a competitive repressor for estrogen receptor-mediated activation of the mouse lactoferrin gene. Mol. Cell. Biol..

[52] Burbach J.P., Lopes da Silva S., Cox J.J., Adan R.A., Cooney A.J., Tsai M.J., Tsai S.Y. (1994). Repression of estrogen-dependent stimulation of the oxytocin gene by chicken ovalbumin upstream promoter transcription factor I. J. Biol. Chem..

[53] Lazennec G., Kern L., Valotaire Y., Salbert G. (1997). The nuclear orphan receptors COUP-TF and ARP-1 positively regulate the trout estrogen receptor gene through enhancing autoregulation. Mol. Cell. Biol..

[54] Petit F.G., Metivier R., Valotaire Y., Pakdel F. (1999). Synergism between a half-site and an imperfect estrogen-responsive element, and cooperation with COUP-TFI are required for estrogen receptor (ER) to achieve a maximal estrogen-stimulation of rainbow trout ER gene. Eur. J. Biochem..

[55] Metivier R., Gay F.A., Hubner M.R., Flouriot G., Salbert G., Gannon F., Kah O., Pakdel F. (2002). Formation of an hER alpha-COUP-TFI complex enhances hER alpha AF-1 through Ser118 phosphorylation by MAPK. EMBO J..

[56] Poulain S., Evenou F., Carre M.C., Corbel S., Vignaud J.M., Martinet N. (2009). Vitamin A/retinoids signalling in the human lung. Lung Cancer.

[57] Kliewer S.A., Umesono K., Heyman R.A., Mangelsdorf D.J., Dyck J.A., Evans R.M. (1992). Retinoid X receptor-COUP-TF interactions modulate retinoic acid signaling. Proc. Natl. Acad. Sci. USA.

[58] Kyakumoto S., Ota M., Sato N. (1999). Inhibition of retinoic acid-inducible transcription by COUP-TFI in human salivary gland adenocarcinoma cell line HSG. Biochem. Cell Biol..

[59] Paez-Pereda M., Kovalovsky D., Hopfner U., Theodoropoulou M., Pagotto U., Uhl E., Losa M., Stalla J., Grubler Y., Missale C., Arzt E., Stalla G.K. (2001). Retinoic acid prevents experimental Cushing syndrome. J. Clin. Invest..

[60] Lin B., Chen G.Q., Xiao D., Kolluri S.K., Cao X., Su H., Zhang X.K. (2000). Orphan receptor COUP-TF is required for induction of retinoic acid receptor beta, growth inhibition, and apoptosis by retinoic acid in cancer cells. Mol. Cell. Biol..

[61] Lin F., Kolluri S.K., Chen G.Q., Zhang X.K. (2002). Regulation of retinoic acid-induced inhibition of AP-1 activity by orphan receptor chicken ovalbumin upstream promoter-transcription factor. J. Biol. Chem..

[62] Lee B.C., Cha K., Avraham S., Avraham H.K. (2004). Microarray analysis of differentially expressed genes associated with human ovarian cancer. Int. J. Oncol..

[63] De Sousa Damiao R., Fujiyama Oshima C.T., Stavale J.N., Goncalves W.J. (2007). Analysis of the expression of estrogen receptor, progesterone receptor and chicken ovalbumin upstream promoter-transcription factor I in ovarian epithelial cancers and normal ovaries. Oncol. Rep..

[64] Ham W.S., Lee J.H., Yu H.S., Choi Y.D. (2008). Expression of chicken ovalbumin upstream promoter-transcription factor I (COUP-TFI) in bladder transitional cell carcinoma. Urology.

[65] Shin S.W., Kwon H.C., Rho M.S., Choi H.J., Kwak J.Y., Park J.I. (2009). Clinical significance of chicken ovalbumin upstream promoter-transcription factor II expression in human colorectal cancer. Oncol. Rep..

[66] Adorno M., Cordenonsi M., Montagner M., Dupont S., Wong C., Hann B., Solari A., Bobisse S., Rondina M.B., Guzzardo V., Parenti A.R., Rosato A., Bicciato S., Balmain A., Piccolo S. (2009). A Mutant-p53/Smad complex opposes p63 to empower TGFbeta-induced metastasis. Cell.

